# Spermatogonial Stem Cells for In Vitro Spermatogenesis and In Vivo Restoration of Fertility

**DOI:** 10.3390/cells9030745

**Published:** 2020-03-18

**Authors:** Fahar Ibtisham, Ali Honaramooz

**Affiliations:** Department of Veterinary Biomedical Sciences, Western College of Veterinary Medicine, University of Saskatchewan, Saskatoon, SK S7N 5B4, Canada; fmi065@mail.usask.ca

**Keywords:** spermatogonial stem cells, germline stem cells, male germ cells, in vitro culture, in vitro spermatogenesis, male infertility

## Abstract

Spermatogonial stem cells (SSCs) are the only adult stem cells capable of passing genes onto the next generation. SSCs also have the potential to provide important knowledge about stem cells in general and to offer critical in vitro and in vivo applications in assisted reproductive technologies. After century-long research, proof-of-principle culture systems have been introduced to support the in vitro differentiation of SSCs from rodent models into haploid male germ cells. Despite recent progress in organotypic testicular tissue culture and two-dimensional or three-dimensional cell culture systems, to achieve complete in vitro spermatogenesis (IVS) using non-rodent species remains challenging. Successful in vitro production of human haploid male germ cells will foster hopes of preserving the fertility potential of prepubertal cancer patients who frequently face infertility due to the gonadotoxic side-effects of cancer treatment. Moreover, the development of optimal systems for IVS would allow designing experiments that are otherwise difficult or impossible to be performed directly in vivo, such as genetic manipulation of germ cells or correction of genetic disorders. This review outlines the recent progress in the use of SSCs for IVS and potential in vivo applications for the restoration of fertility.

## 1. Introduction

Spermatogonial stem cells (SSCs) are a small group of testis cells, residing at the basal membrane of seminiferous tubules. They can undergo measured mitotic divisions to balance self-renewal and formation of exponentially increasing numbers of differentiating germ cells. What makes SSCs unique among all other adult stem cells is their potential for contributing genes to the next generation. Due to their fundamental role in maintaining spermatogenesis, SSCs ensure a supply of millions to billions of sperm per day throughout the reproductive life of a male. SSCs produce cohorts of daughter germ cells to undergo synchronized, sequential, and extensive differentiation processes to be transformed from a typical spherical cell into self-propelling vehicle systems with only one mission, to deliver the male haplotype into the female counterpart, the haploid oocyte [1,2]. Considerable insight has been gained in the past three decades about the functional potential of SSCs in initiating spermatogenesis, and even their potential to be driven into pluripotency for use as an alternative to embryonic stem (ES) cells. However, we are still far from sufficient understanding of SSCs and realizing their full potential in assisted reproductive technologies and stem cell therapy. This is largely due the difficulty of unequivocal identification of SSCs and the complexity of replicating their differentiation properties and function in vitro [3,4].

Since SSCs are very rare in the testis, development of effective and efficient in vitro culture systems that support the maintenance and expansion of SSCs is crucial for their characterization and manipulation. Moreover, optimal conditions for the in vitro culture and propagation of SSCs are also needed to help boost the potential therapeutic application of SSCs in fertility preservation or restoration. Long-term culture of rodent SSCs is well demonstrated; however, relatively few in vitro culture conditions have been examined for primate SSCs [2]. Any potential in vivo application of cultured human SSCs, such as in restoration of fertility, requires extensive studies to ensure their safety and efficacy. The establishment of efficient culture conditions for human SSCs also necessitates the availability of proper animal models, for instance, xenotransplantation techniques to assess the quality and quantity of such cultured SSCs; these assays have so far been used sporadically for testing primate SSCs [4].

Spermatogenesis is a complex process of germ cell proliferation and differentiation that requires extensive interactions among different cell types, hormones, growth factors, and various other signals, making it difficult to be replicated in vitro. There are several objectives for establishing an optimal and efficient culture system to recapitulate the process of germ cell development in vitro. This includes the study of basic requirements of male germ cell development, proliferation, differentiation, and production of haploid germ cells in a controlled in vitro environment. Additionally, such culture systems could be potentially used to produce haploid male germ cells from undifferentiated germ cells isolated from the testis of infertile adult patients and/or testicular biopsies collected from prepubertal cancer patients before undergoing gonadotoxic treatment. The establishment of culture systems for in vitro spermatogenesis (IVS) would also enable experimentations that are otherwise difficult to be performed directly in vivo such as pharmaceutical or toxicological study of new drugs or potential toxicants on human spermatogenesis. Other applications include the study of mechanisms of testicular tumors, genetic causes of male infertility, or even correction of genetic disorders causing infertility. Current approaches to IVS can be divided into three general categories including organ/tissue culture, and two-dimensional (2D) or three-dimensional (3D) cell suspension culture systems. Moreover, developing an efficient IVS model can be of benefit not only from a research perspective but also from an animal ethics point of view by using non-animal models.

This article summarizes the progress made in offering efficient methods for SSC isolation, characterization, in vitro culture, and in vitro differentiation, particularly using primate models. Moreover, in this article we review some of the salient proposed approaches for the preservation and restoration of fertility in prepubertal and pubertal patients using currently-available and potential future SSC-driven biotechnological strategies.

## 2. Spermatogonial Stem Cells (SSCs)

Male germline stem cells include SSCs, as a better-known component, as well as two earlier cellular stages, namely primordial germ cells (PGCs) and gonocytes. All mammalian germ cells originate from PGCs which are the primary cells of the germline lineage in both male and female embryos. PGCs first appear as a small population of alkaline phosphatase-expressing cells at ~7.5–8 days post-coitum (dpc) in rodents and at ~three weeks gestation in humans. Drawn by chemotaxis, PGCs migrate to the genital ridge and reside in the indifferent gonad at ~11–13 dpc and 4–5 weeks gestation in rodent and human embryos, respectively. Once PGCs lose alkaline phosphatase expression, they become known as gonocytes at ~14.5 dpc in rodents and ~seven weeks gestation in human embryos. After birth, gonocytes transform into spermatogonia at ~5 days post-partum (dpp) in rodents and ~three months after birth in newborn boys [5,6].

**Figure 1:**
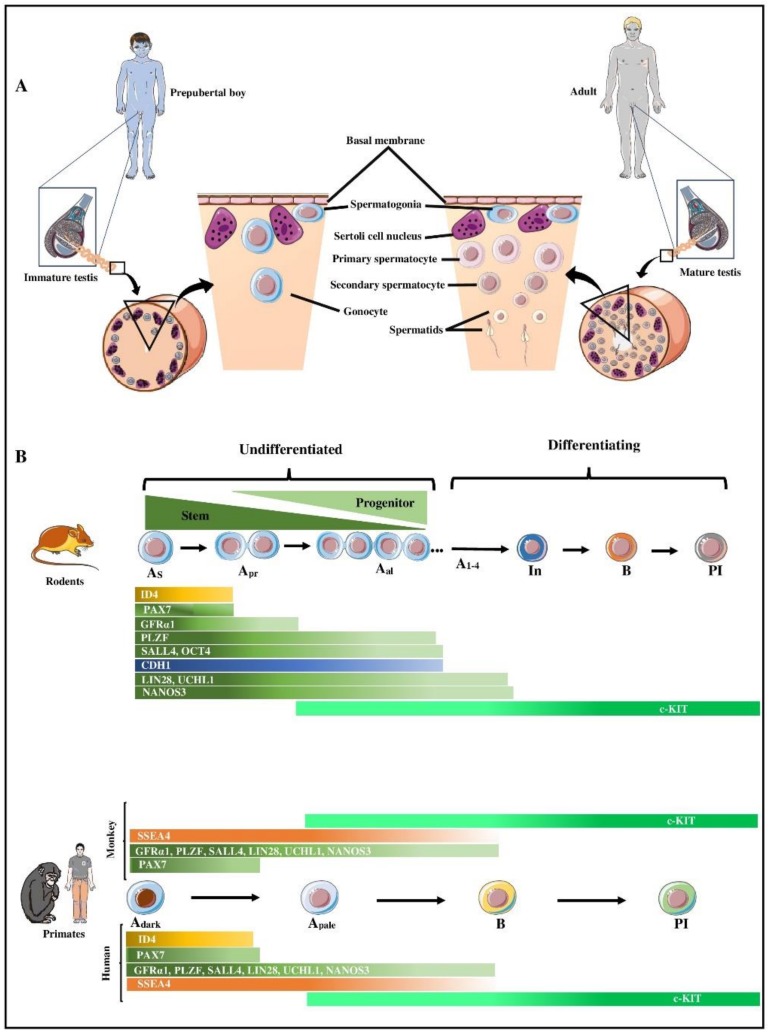
Schematic representation of various spermatogenic cells present in the prepubertal and adult testis, as well as comparative representation of spermatogonial markers in rodents and primates. (**A**) A schematic pie slice of a seminiferous cord/tubule of a human prepubertal and an adult showing the different types of spermatogenic cells present. Gonocytes or spermatogonia (depending on age) are the only types of germ cells present in the testis of prepubertal boys, whereas in the adult testis all stages of spermatogenic cells can be present. Spermatogonia reside at the basal membrane and are intra-tubularly surrounded by Sertoli cells. Spermatogonia undergo differentiation into spermatocytes, spermatids, and ultimately sperm. (**B**) Comparative developmental patterns and expression profile of select spermatogonial markers in rodents and primates. Rodents and primates have several common spermatogonial markers (green color-coded) with few exceptions (blue, orange, and gold-coded). For a more complete list of the currently-known molecular markers of rodent and primate spermatogonia see Table 1. A_s_: Type A-single spermatogonia; A_pr_: Type A-paired spermatogonia; A_al_: Type A-aligned spermatogonia; A_dark_: Type A spermatogonia with dark nuclei; A_pale_: Type A spermatogonia with pale nuclei; In: intermediate spermatogonia; B: Type B spermatogonia; Pl: preleptotene spermatocytes.

As depicted in Figure 1A, parenchyma of the postnatal testis tissue has a compartmentalized structure where germ cells along with their main supporting somatic, Sertoli, cells are enclosed in seminiferous tubules (or cords when they lack a lumen in immature testes), surrounded by peritubular myoid cells. The space between the tubules/cords is occupied by androgen producing Leydig cells, along with other components of the connective tissue. Spermatogenesis involves various interactions between somatic cells and germ cells. The process of spermatogenesis begins with spermatogonia that reside at the basal membrane which undergo a proliferation phase, comprised of a series of mitotic divisions to exponentially increase the number of germ cells, before undergoing meiosis to be transformed into haploid germ cells, spermatids and sperm. This process is repeated regularly at pre-determined intervals to ensure a continuous supply of sperm is present at any given time during the reproductive life of an adult male. Based on morphological studies using rodent models, spermatogonia in non-primate mammals have been proposed to include stem cell (A_single_), proliferative (A_paired_, A_aligned_), and differentiating (A_1_, A_2_, A_3_, A_4_, intermediate, and B) spermatogonia.

Type A_single_ spermatogonia are present as individual half-moon-shaped cells in close proximity to the basal membrane [1,7]. Being the most primitive spermatogonia, A_single_ spermatogonia are believed to be SSCs which can undergo a symmetric division to produce two separate, but otherwise similar, self-renewing A_single_ (SSCs) to increase their numbers and form a stem cell pool, for instance to regenerate steady-state spermatogenesis after cytotoxic insult. Alternatively, and during steady-state spermatogenesis, SSCs can undergo an asymmetric division to produce a self-renewing A_single_ to maintain the stem cell pool and a progenitor A_single_, destined for differentiation. Each progenitor A_single_ can then divide to produce two cells which remain connected, hence referred to as A_paired_ spermatogonia [8]. All subsequent generations of germ cells also remain connected through intercellular cytoplasmic bridges, thought to be important for the coordination of their progression in the differentiation process. Hence, clones of 4-16 or sometimes 32 germ cells are formed in a chain, known as A_aligned_ spermatogonia [1,7].

Type A spermatogonia that belong to the A_single_, A_paired_, and A_aligned_ stages are collectively referred to as undifferentiated spermatogonia, but transplantation studies have concluded that they are indeed a heterogenous population. An adult mouse testis contains ~35,000 A_single_ out of a total number of ~330,000 undifferentiated spermatogonia. Although A_single_ are typically designated as ‘true’ SSCs, functional assays have shown that only an estimated 8.5%–17% of all A_single_ have stem cell potential. In other words, >80% of A_single_ are in fact not SSCs; however, it has been suggested that stem cell activity is strongest but not restricted to A_single_. Although an exact point of no-return has not been determined, it is believed that the SSC activity sharply declines as spermatogonia progress into A_paired_, and especially into A_aligned_. The same functional assays conclude that only ~3000–6000 ‘functional’ SSCs exist in an adult mouse testis, comprising 0.9%–1.8% of all undifferentiated spermatogonia or 0.01%–0.02% of total cells in the seminiferous tubules [9].

Larger clones of A_aligned_ spermatogonia differentiate without a cellular division to A_1_ spermatogonia and initiate synchronized further mitotic divisions to produce types A_2_, A_3_, A_4_, intermediate, and B spermatogonia. While under conventional histological preparations various undifferentiated spermatogonia show similar cell-nuclear morphology, transition of A_aligned_ into A_1_ signals the start of differentiation and involves major changes in morphology and mitotic behavior. Type B spermatogonia must physically pass through the Sertoli-cell barrier (also known as the blood-testis barrier), before dividing to produce primary spermatocytes which in turn undergo two meiotic divisions to give rise to secondary spermatocytes and round spermatids, before undergoing spermiogenesis to produce sperm [4,7,9,10].

In primate testes, categorization of spermatogonia also includes type A and B spermatogonia but especially for type A spermatogonia it differs and includes A_dark_ and A_pale_. Designation as A_dark_ is primarily based on their dark nuclear staining with hematoxylin. A_dark_ are believed to comprise a population of ‘reserve’ stem cells since they show low proliferative activity during normal spermatogenic activity. In contrast, A_pale_ are less densely stained during histological processing and are thought to proliferate continuously. Although, both A_dark_ and A_pale_ reside at the basal membrane, they have other morphological differences; A_dark_ are characterized as being relatively small, round or slightly ovoid, while A_pale_ are relatively larger, oval or almost round cells. Subpopulations of A_dark_ have been shown to contain 1–3 chromatin-free vacuolar spaces known as nuclear rarefaction zones [11], which can be used as a background morphological indicator to help discriminate spermatogonial subtypes undergoing immunohistochemical analyses.

In 1959, Clermont proposed that A_dark_ are SSCs which undergo self-renewal to maintain their population and at the same time also give rise to A_pale_ that subsequently generate differentiating B spermatogonia. Ten years later, Clermont revised his model based on the incorporation of 3H-thymidine in A_pale_ instead of A_dark_ spermatogonia of vervet monkeys, suggesting that A_pale_ are the ‘active’ stem cells that maintain spermatogenesis in the adult testis [12]. In humans, a similar model has been proposed, where A_pale_ act as active stem cells to maintain their population and produce differentiating B spermatogonia. However, some believe that the original model was more accurate, stating that A_dark_ are indeed true SSC because of their low mitotic index, while nearly all A_pale_ undergo regular divisions suggesting that these cells are the ‘renewing progenitors’ that amplify spermatogonial output to B1. Primate A_pale_ and A_dark_ spermatogonia are considered as counterparts of rodent undifferentiated spermatogonia (A_single_, A_paired_, and A_aligned_), and primate type B spermatogonia as equivalents of rodent differentiating spermatogonia (A_1_–A_4_, intermediate and B spermatogonia) (Figure 1B). Interestingly, the rhesus monkey testis contains approximately the same total number of A_dark_ and A_pale_ as in the human [13,14].

In humans, A_pale_ divide every 16 days, the length of one seminiferous epithelium cycle, and repeat it ~4.5 times; hence the germ cells that resulted from the first division of A_pale_ become sperm after ~75 days [15]. A low proliferative activity of SSCs is deemed beneficial [16], because it decreases the chance of errors in DNA duplication during the S phase. While the number of spermatogonial divisions from SSCs to spermatocytes is comparable between the monkey and non-primates, this number is lower in humans compared with many non-primates. In humans, there is only one spermatogonial generation between the A_1_ and B stages [17], while there are six divisions separating these stages in most non-primate mammals [18].

## 3. Characteristics of Spermatogonia in Primates

From the above discussion it can be concluded that SSCs are a rare and heterogenous subpopulation of undifferentiated spermatogonia that can only be defined by their function, not morphology. Hence, there is a need to identify specific biomarkers that allow isolation and propagation of SSCs to better gain insight into their biology and harness their potential. In response to this need, in 1994 using mouse models, a technique for transplantation of germ cells was developed which can be viewed as a unique bioassay to assess the stem cell activity of putative SSCs in any given population of testis cells.

Germ cell transplantation involves the microinjection of testis cells from a donor male into the seminiferous tubules of a recipient male. This transplantation technique has also been extended to various species, but performing primate-to-primate germ cell transplantation is more technically challenging. Although, primate-to-mouse germ cell transplantation has been shown to allow evaluation of primate spermatogonia, it provides limited information regarding the identity of primates SSCs. Nevertheless, this assay has ever since been used as a routine method for the identification, quantification, characterization, and confirmation of functional competence of SSCs. Our recent knowledge of the molecular signature and biomarkers of SSCs, especially in rodents, has allowed enrichment of testis cells for SSCs to tens- or even hundreds of folds; however, still no unequivocal and exclusive SSC marker is available. Therefore, identification of SSC markers in primates remains as one of the areas of intense research in male reproductive science and medicine [4,19,20,21].

Analysis of monkey spermatogonia demonstrated that they share certain germ cell and SSC markers with rodent spermatogonia (Figure 1B, Table 1). Some of these markers are expressed on the cell surface and hence can be potentially used for isolation and enrichment through cell sorting methods, such as fluorescence-activated cell sorting (FACS) or magnetic-activated cell sorting (MACS). Other spermatogonial markers are located intracellularly and can only be used for retrospective identification of SSCs.

Mouse gonocytes and spermatogonia express LIN28 throughout testicular development [22]. The analysis of newborn macaques (*Macaca mulatta)* testes also revealed that 100% of seminiferous tubules contained LIN28^+^ germ cells; however, the number of immune-positive cells decreased during testicular development. On the other hand, although several LIN28^+^ tubular cross-sections in adult mouse testes can be detected, only a few LIN28^+^ tubular cross-sections can be observed in adult marmoset, rhesus, or human testes [23]. Marmoset gonocytes and spermatogonia have also shown expression for SALL4, where the proportion of SALL4^+^ germ cells decreased during puberty and was restricted to A_dark_ and A_pale_ spermatogonia in pubertal and adult testes. The expression of SALL4 was demonstrated in the majority of gonocytes in fetal human testes and type A spermatogonia of 1-year-old boys [24]. Adult rhesus testicular tissues also showed the expression of DDX4 (VASA), DAZL, GFRα1, and PLZF [25]. Interestingly, the number of PLZF^+^ cells was calculated to be ~1.86 per cross-section, suggesting that the SSC population in monkey testes is a subset of either the A_dark_ or A_pale_ spermatogonia. Other known markers of non-human primate spermatogonia include DPPA4 [26], TRA-1-60, TRA-1-81, [27], and THY1 [28].

Similar to non-human primates, spermatogonia and their progenitors in humans and rodents also share some but not all markers (Figure 1B, Table 1). For instance, in mice, α6-integrin, β1-integrin [29], and THY1(CD90) [30] are well-known surface markers of SSCs/progenitor cells, while CD9 is a surface marker of both rat and mouse SSCs [31]. Surface markers including α6-integrin, CD90, GFRα1, and CD133 have also been successfully used to select human spermatogonia using MACS [32]. The expression of PLZF has also been observed in whole mounts of seminiferous tubules of human testes [33]. Additionally, ID4 [34] and GPR125 are considered markers for mouse spermatogonia and their progenitors [35], and their expression has also been observed in human spermatogonia [36]. In contrast, some other markers of rodent spermatogonia and their progenitors may not be conserved in humans. For example, it remains to be explored whether certain markers of rodent spermatogonia such as RET [37], STRA8 [38], CDH1 [39], and NEUROG3 (NGN3) [40] are also present in human spermatogonia. Conversely, certain specific markers of human spermatogonia have not been observed in rodents. For example, TSPY, a specific marker for human spermatogonia [41] is not expressed by rodent spermatogonia; however, elongated spermatids of rats are positive for TSPY [42]. Similarly, other markers of human spermatogonia such as CD133 [32] or CHK2 [43] are yet to be examined for expression in rodents. Such studies can further reveal similarities and differences between spermatogonia in rodents and primates.

## 4. Isolation and Enrichment of SSCs in Primates

Because SSCs are a very rare subpopulation of testis cells, their use in downstream applications requires optimal isolation and purification, as an important first step. In addition to the need for large numbers of SSCs for applications such as transplantation into recipient testes, access to additional SSCs is also warranted for critical analysis of cultured cells in terms of genetic and epigenetic stability as well as functionality.

Testis cells can be isolated using enzymatic digestion, usually involving the use of a combination of enzymes in two steps. In brief, after removing the tunica albuginea and excess connective tissue, the testis parenchyma is divided into smaller fragments to be first incubated with collagenase to disperse the seminiferous tubules, followed by the addition of trypsin to obtain a single-cell suspension. If necessary, DNase-I is also added to prevent adhesion of the resultant cells. Two-step enzymatic digestion protocols have been widely used for digestion of testis tissue from non-human primates [44] and humans [75]. Since this digestion method is not an optimized or cell-targeted process, it generates a mixed population of testis cells where SSCs are present at low numbers. Therefore, it would be ideal if modified protocols were to be developed for digestion of primate testis tissue to favor the isolation toward collecting more germ cells. This optimization can be similar to a three-step digestion protocol that we developed specifically for neonatal pig testes which yields ~40% highly-viable gonocytes within an hour [76]. In the absence of optimized digestion protocols, the obtained testis cell suspensions would typically need to go through further enrichment steps to reduce the number of contaminating somatic cells or advanced stages of germ cells.

Enrichment of germ cells can be achieved with or without the use of antibodies. Specific antibodies may be used to separate SSCs (positive selection) or to exclude contaminating cells (negative selection) using FACS or MACS. For instance, MACS with an anti-GFRα1 antibody has been used for the isolation of spermatogonial subpopulations from adult monkey and human testes. Gassei et al. (2010) reported that about two thirds of GFRα1-positive cells found in the sorted fractions resembled A_dark_ spermatogonia, while one third resembled the A_pale_ [77], suggesting that most of the isolated cells were SSCs. MACS has also been successfully employed for the enrichment of human spermatogonia using antibodies against GPR125 [33] and SSEA4 [48]. Nickkholgh et al. (2014) concluded that MACS for ITGA6^+^ and HLA^−^/GPR125^+^ can be used for efficient enrichment of human spermatogonia [78]. The use of FACS to select OCT4-positive cells has also resulted in obtaining 87% purity of human SSCs [79]. Compared to FACS, MACS is considered a more efficient, accurate, and easier method for enrichment of SSCs, especially since it does not require large numbers of cells. Thus, MACS is better suited for applications such as isolating SSCs from cells that result from small biopsies of human testis tissue.

Germ cell enrichment strategies that do not implement antibodies rely on the innate cellular characteristics that differentiate germ cells from somatic cells. Such strategies include the affinity of different cell types for adhesion to the culture plate or to extracellular matrices (ECM). Since Sertoli cells adhere to the culture plate faster than SSCs, differential planting is a widely-used technique for the separation of germ cells from somatic cells. Alternatively, differences in velocity sedimentation or density gradient centrifugation can be used to separate somatic and germ cells. Percoll density gradient for instance has been used for cell separation of human testis cells leading to ~87% pure population of SSCs [79]. However, adjusting the timing for cell sedimentation is critical to prevent the loss of large numbers of SSCs during the high activity of the sedimentation process [80].

## 5. In Vitro Culture of Primate Gonocytes/Spermatogonia

Culture of rodent gonocytes/spermatogonia is well demonstrated [81]; however, especially long-term culture and expansion of primate SSCs, and conclusive demonstration of their SSC potential has been challenging. Establishment of an efficient in vitro culture system to maintain both the self-renewal and proliferation capacity of human SSCs is crucial for their potential clinical applications. Culturing human SSCs, isolated from testicular biopsies of obstructive azoospermia patients (aged 22–35 years), in StemPro-34 SFM (serum free medium) containing growth factors (commonly referred to as a serum-free germline culture medium) and hydrogel, resulted in significant propagation of SSCs for two months. During the culture, expression of numerous SSC markers was maintained, suggesting that SSCs retained their undifferentiated status [82]. Moreover, similar culture conditions appear to also support the short-term in vitro culture of human SSCs from healthy individuals without altering their undifferentiated status [33].

The media initially used to culture human hematopoietic stem cells also supported the long-term culture of human SSCs [83]. The growth factors used in the latter study (i.e., GDNF, BFGF, EGF, and LIF) were previously reported to support the long-term culture of rodents SSCs [84]. In these culture conditions, human testicular cells formed two types of cell colonies; one had a flat morphology and an appearance similar to ES cells, while the other had rounded morphology. Morphologically, round cells showed an expression profile typical of spermatogonia (e.g., PLZF, ITGA6, and ITGB). The SSC clusters presented as clumps of individually visible cells, while colonies of ES-like cells were sharp-edged and compact. In these culture conditions, human SSCs could be propagated for 15 weeks, after which germline stem cell clusters no longer appeared, and ES-like cells and somatic cells started detaching from the dish. It was also found that sub-culturing human SSCs under feeder cell-free conditions in laminin-coated culture dishes could increase the duration of propagation (up to 20 weeks). Xenotransplantation assays showed an impressive 18,450-fold increase in human SSC numbers within a time frame of 64 days [83].

The germline culture medium also supported the short-term in vitro culture of non-human primate SSCs [24], while Stem-Pro medium, supplemented with four growth factors (i.e., bFGF, GDNF, LIF, and EGF), actively maintained the SSCs isolated from prepubertal cynomolgus monkeys (aged 44–57 months) [85]. However, the lack of using functional assays to verify human SSCs in most studies, and similarities between SSC markers of human and rodents in other studies make the results controversial. Moreover, many markers used to identify human SSCs (e.g., PLZF, GFRA1, UCHL1, GPR125, and ITGA6) can also be observed in many testicular somatic cells, suggesting that these markers are not reliable. The only dependable protocol to access whether cultured SSCs are indeed functional germ cells is homologous transplantation of in vitro-cultured SSCs into a recipient testis (of another male of the same species) and subsequent generation of offspring. However, due to obvious ethical concerns, this approach is not practical in humans and difficult in monkeys. In non-human primates, different stage-specific germ cell markers have been better-defined compared with humans. Therefore, to validate the true nature of germ cells in the culture of non-human primates, monitoring of validated germ cell markers can be used to establish an efficient in vitro culture condition. Development of a definitive functional assay to evaluate the competency of human SSCs or to identify reliable SSC-specific markers would facilitate finding a culture condition for long-term in vitro maintenance of SSCs.

## 6. In Vitro Models of SSC Differentiation

Establishing efficient in vitro culture systems that can replicate the process of male germ cell development and spermatogenesis has several important applications. These applications include the study of basic requirements, mechanisms of action, and cell-to-cell interactions of male germ cells during development, proliferation, differentiation, and production of haploid germ cells in a controlled in vitro environment. For instance, the use of germ cells from infertile patients may help investigations into the various causes of male infertility due to stage-specific blockage of germ cell differentiation. Development of efficient culture systems for IVS would also allow experimentations that are otherwise difficult to be performed directly in vivo, such as genome editing of germ cells or correction of genetic causes of infertility. Such models can be of benefit not only from a research perspective but also from an animal ethics view by using non-animal models. Various research groups have taken different routes toward the goal of developing an in vitro culture system; these can be classified into one of three general approaches, namely organotypic culture of testicular tissue fragments, and two-dimensional or three-dimensional culture of testis cell suspensions.

### 6.1. Organotypic Culture of Testicular Tissue Fragments

The unique three-dimensional structure of seminiferous cords/tubules seems essential in facilitating the required interactions between germ cells and somatic cells. This partly includes providing a proper niche for SSCs to reside, and establish or maintain spermatogenesis. Thus, it makes sense to first try to culture small fragments of testis tissue or intact pieces of semiserious tubules; indeed, this is what many of the pioneering groups have done in the past and many current research labs are pursing. Organotypic culture of tissues and organs is, however, challenging because of the issues arising from the limited diffusion rates of the tissue, especially compared with monolayer cell cultures. As a result, a main hurdle in achieving IVS using testicular tissue fragments is maintenance of the tissue viability for the required length of culture.

In 1959, Trowell designed an organ culture system by employing a gas-liquid interface in which testicular fragments from adult rats could be maintained in Eagle’s minimum essential media (MEM) at 37 °C with 5% CO_2_ in air, albeit only for 6 days [86]. In 1964, another group using the same organ culture system was able to maintain testicular fragments of 4-day-old rats viable for four weeks, although cell differentiation was not observed [87], but in the same year they also reported differentiation of spermatogonia to spermatocytes within 2-3 weeks of culture [88]. The effects of temperature, pH, vitamins, hormones, sera, and tissue extracts were also examined; however, developmental progress was still limited up to pachytene spermatocyte [89]. These latter culture conditions were also used to maintain human testicular tissues in vitro for several weeks [90]. It was then shown that FSH is required for germline differentiation and more specifically in the conversion of type A spermatogonia into meiotic pachytene spermatocytes [91]. In 2003, Suzuki and Sato used a gas-liquid interface culture system that was developed decades earlier [92] with minimal changes to culture 5-day-old mouse testis tissue, but interestingly, round spermatids could be observed after two weeks of culture [93].

A dramatic turn in the long-standing efforts for replicating complete IVS took place when in 2011, Sato et al. reported successful production of offspring from in vitro produced haploid germ cells [94]. Testicular fragments of neonatal mice were cultured on agarose gel (half-socked in medium) in α-MEM supplemented with knockout serum replacement (KSR) or 40 mg/mL AlbuMAX instead of fetal bovine serum (FBS). Interestingly, this culture system was also successful for in vitro production of haploid male germ cells from the testis tissue of an infertile mutant mouse model [95] as well as after cryopreservation of the testis tissue [96]. The agarose gel-based organ culture system also led to the production of haploid male germ cells from adult mouse donors; however, the efficiency was far lower than that from neonatal donor mice [97].

**Figure 2:**
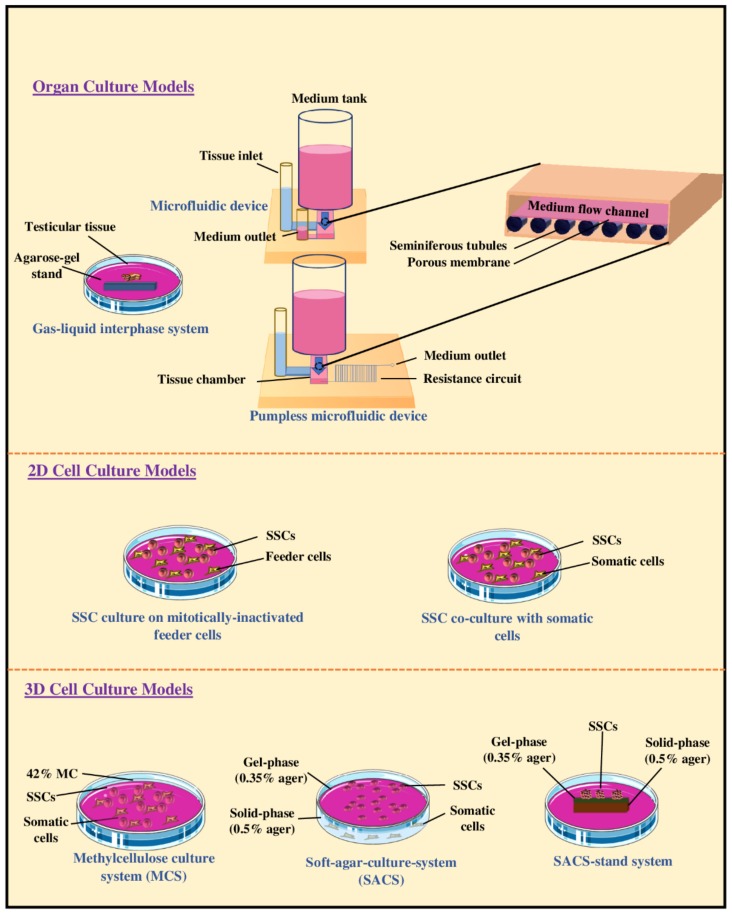
Schematic representation of organotypic culture of testicular tissue fragments, two-dimensional (2D), or three-dimensional (3D) cell suspension culture models for in vitro spermatogenesis. Three approaches to organotypic culture of testicular tissue fragments have been proven effective including using a gas-liquid interface system, a conventional microfluidic system, or a pumpless microfluidic device. 2D culture of cell suspensions can be achieved through two approaches, namely culturing isolated SSCs on mitotically-inactivated feeder cells (e.g., mouse embryonic fibroblast cells) or culturing on mixed populations of testicular cells (co-culturing of germ cells and somatic cells). 3D cell cultures can include using methylcellulose (MCS), a soft-agar culture system (SACS), or a SACS-stand system.

Subsequently, to improve the efficiency and duration of in vitro spermatogenesis (IVS), the agarose gel-based organ culture system was modified and a microfluidic technology was adopted for organ culture systems (Figure 2). The main rationale for using a microfluidic system was to encourage the exchange of substances such as gases, nutrients, and waste products by facilitating diffusion and creating conditions that are more representative of in vivo. Surprisingly, the microfluidic device allowed the maintenance of mouse testicular tissue for ~six months, prompted the induction of spermatogenesis, and led to the in vitro production of spermatids and sperm capable of giving rise to offspring after micro-insemination [98]. Ever since, pumpless microfluidic devices have also been developed (Figure 2). These systems use hydrostatic pressure and a resistance circuit to facilitate slow flow of the medium; this has led to the induction of efficient IVS for ~three months [99]. Taken together, these results suggest that the organotypic culture of testicular tissue or fragments is capable of maintaining the architecture and viability of germ cells, and induction of IVS. Moreover, the addition of a microfluidic device has shown the potential to improve organotypic culture systems, as it can lead to long-term ex vivo maintenance of testis tissues which is required for producing sperm.

### 6.2. Two-Dimensional Culture of Testis Cell Suspensions

Two-dimensional (2D) culture systems using enzymatically-dispersed testis cell suspensions have also been widely used for both in vitro proliferation of SSCs and production of haploid male germ cells (Table 2, Figure 2). Compared with organotypic cultures of testicular tissue, the 2D culture systems for testis cell suspensions have innate advantages and disadvantages. A 2D cell culture system can be potentially used to expand SSC numbers in vitro for downstream applications such as in auto-transplantation to the individual’s testis following recovery from cancer. Using 2D culture systems of testis cells is also ideal when the objective is to investigate the role of individual cell types, putative factors, or candidate genes or gene-products on fate of germ cells. However, compared with organotypic testicular tissue culture systems, 2D culture of cells has at least two disadvantages. Firstly, it does not resemble or replicate the expected cellular interactions and structural conditions of intact seminiferous tubules, and secondly, in the absence of spatial positioning and typical cellular morphology, it is difficult to distinguish different types of cells without further analyses such as the use of flow cytometry.

In 1983, it was reported that germ cells isolated from 6–15 wk-old mice cultured using a 2D system were able to differentiate into spermatocytes [100]. Interestingly, in 1989, haploid male germ cells were produced from testis cell suspensions of 14-day-old rats cultured on Type 1 collagen gels in a 1:1 mixture of Ham’s F12 medium and Leibovitz’s L15 medium with 10% FBS, epinephrine, and norepinephrine [101]. In 1993, there were two reports providing evidence that immature germ cells can be directed to form haploid male germ cells using 2D culture systems [102]; however, no follow-up studies using the same protocol have been published. In 1998, Durand’s group reported that they have produced haploid male germ cells from single-cell preparations of 23–25 day-old rats cultured in DMEM-F12 supplemented with 0.2% FBS, testosterone, and FSH [103]. They attributed their success to maintaining maximal interactions between Sertoli cells and spermatogenic cells. This raised awareness among researchers as to the importance of germ cell-Sertoli cell interactions and other groups tried to maintain such cellular interactions to encourage spermatogenesis in vitro. In 2000, the same group reported further in vitro differentiation of 2D cultured isolated advanced germ cells (i.e., leptotene spermatocytes), collected from 20, 22, or 28-day-old rats to haploid male germ cells [104].

The first report of successful production of offspring from in vitro produced early-stage haploid male germ cells (i.e., round spermatids) came in 2003 [105]. Germ cells isolated from 13–18 day-old mice were cultured with Sertoli cells in a serum-free, hormone/growth factor-supplemented medium, which after 7–10 days, led to the appearance of fertilization-competent round spermatids from pre-existing spermatocytes [105]. In 2006, the importance of cell contacts between germ cells and feeder cells for IVS was highlighted, when type A spermatogonia of an immature rat (7-day-old) co-cultured with Sertoli cells differentiated to spermatids [106]. More recently, different approaches have been proposed that could direct the differentiation of SSCs all the way to haploid male germ cells. Using isolated 6-day-old mouse SSCs, production of haploid germ cells was achieved by culturing testicular cells in 10% FBS-supplemented media for 3 days, changing the media into a retinoic acid (RA)-enriched medium to induce differentiation for 2 days, and followed by changing the media again to the first medium for 6–8 days [107]. 

In conclusion, even a 2D cell suspension of testis cells under optimal culture conditions can induce spermatogenic differentiation; however, for this to work, cell-to-cell contact between germ cells and feeder cells appears to be important. Although, intensive research over the years has finally led to the emergence of culture conditions suitable for IVS of rodents, considerable gaps in knowledge remain in our understanding of the developmental processes involved.

### 6.3. Three-Dimensional Culture of Testis Cell Suspensions

The knowledge gained through intensive research on organotypic and 2D cell culture systems directed some of the research on testis cell suspensions toward the development of artificially constructed three-dimensional (3D) structures. In 2006, testicular cells isolated from 18-day-old rats were cultured on collagen gels to mimic the composition of basal membrane of seminiferous tubules. Not only did this 3D culture system increase the viability of testicular cells but it also directed the differentiation of germ cells, suggesting that cell-to-cell interactions in a 3D culture system sufficiently support the differentiation of germ cells [113]. In 2008, a soft-agar culture system (SACS) was introduced for IVS (Figure 2), which consisted of two phases based on agar concentrations; a gel phase containing enriched SSCs, and a solid phase comprising supporting cells (e.g., Sertoli cells). The expression of meiotic stage-specific markers was observed when testicular cells obtained from 10-day-old mice were mixed with the gel-agar medium (0.35%) and incubated on a solid-agar base (0.5%) [114]. In 2012, the production of haploid germ cells from undifferentiated germ cells, isolated from 7-day-old mice, was reported using the 3D SACS; however, the fertility of in vitro produced mature germ cells was not determined [115]. This latter condition also directed the differentiation of undifferentiated germ cells isolated from 13–33 month-old rhesus monkeys; an age range corresponding to the immature through pubertal and young adulthood ages [116]. A methylcellulose culture system (MCS) has also been proposed as yet another 3D culture system which appears to support the differentiation of immature germ cells into haploid male germ cells [117].

In order to artificially reproduce the in vivo form and function of the seminiferous epithelium, a 3D engineered blood-testis barrier (eBTB) system was introduced in 2010. In essence, the eBTB is aimed at providing a condition that can maintain the interactions between somatic and germ cells, deemed crucial in achieving spermatogenic differentiation. Testicular peritubular myoid cells were first cultured on the underside of culture inserts 3 days prior to adding a mixture of Sertoli and germ cells on top of the inserts, which after an additional 22 days of culture led to the formation of haploid male germ cells [118]. Yokonishi et al. (2013) reported that under these new 3D culture conditions, dissociated immature testicular cells reconstructed into structures resembling their original tissue architecture. Their new system included using testicular cells isolated from neonatal mice to be cultured in V-shaped plate wells for 2 days to allow aggregate formation, followed by placing the aggregates on top of agarose gel blocks. Interestingly, the aggregated cells formed tubule-like structures, and haploid germ cells were observed after 30–51 days of incubation [119]. Recently, a modified 3D culture method, three-layer gradient system (3-LGS), was also introduced for in vitro generation of testicular organoids using rat testis cell suspensions. In this approach, rat testis cells were suspended in Matrigel and placed between two cell-free layers of Matrigel, which after 7 days led to the formation of testicular organoids. Additionally, self-organization of testicular cells also led to eBTB formation and Sertoli cell epithelization; however, complete differentiation of germ cells was not observed [120]. These results collective suggest that maintaining the microenvironment of testicular cells even in the form of a 3D-culture system is crucial in achieving spermatogenesis ex vivo.

## 7. Progress of In Vitro Spermatogenesis in Primates

The first attempt at achieving IVS using human testicular biopsies was reported in 1967, in which after three weeks of culture, differentiation of primary spermatocytes from the preleptotene to pachytene stage was observed [121]. Later in 1971, testicular tissues obtained by orchidectomy due to prostate cancer were cultured, and round spermatids were the most advanced germ cells observed in the supernatant of the media used for culturing the tissue samples at 27 day of culture [90]. However, in 1981, it was reported that the latter culture system does not support complete differentiation of spermatogonia, rather only cells already committed to meiosis at the time of culture initiation can proceed through spermatogenesis [122].

The limited success of organotypic culture systems using human testis tissue in achieving IVS diverted some of the research focus toward the use of testicular cell suspensions for in vitro differentiation of germ cells. Culturing spermatids, isolated from testicular biopsies of obstructive azoospermia patients, in a culture medium containing FSH showed evidence of maturational process in the form of morphological changes in the spermatid nuclei and flagellar growth after only 48 h [123]. These results presented new hopes for treating infertilities related to germ cell maturation arrest. A year later, when in 1999, spermatids isolated from non-obstructive azoospermia patients were cultured on Vero cells for 5 days, round spermatids differentiated into elongated spermatids and a single mature spermatozoon was also observed [124]. Vero cell-conditioned medium supplemented with hormones also supported the in vitro meiosis and spermiogenesis of co-cultured somatic and germ cells isolated from testicular biopsies of non-obstructive azoospermic patients [125]. In 2003, the differentiation efficiency of primary spermatocytes, from azoospermic men with spermatogenic arrest, was tested after co-culture with Vero cells using various culture conditions. Primary spermatocytes co-cultured with Vero cells in MEM-based media, supplemented with 50% boar rete testicular fluid or in human synthetic oviduct fluid and 10% human serum, resulted in the generation of round spermatids at a rate of 10%. Moreover, the presence of 23 chromosomes and chromatids confirmed that the in vitro produced cells were indeed haploid [126]. In 2012, SSCs isolated from testicular biopsies of obstructive and nonobstructive azoospermic patients were co-cultured with Sertoli cells in a KSR-supplemented medium for 5 days, when fluorescence in situ hybridization (FISH) analysis of chromosomes confirmed the presence of haploid male germ cells [127]. However, a 5-day period of culture seems far too short to allow completion of the complex developmental process of differentiation from primary spermatocytes to round spermatids.

More recently, using an organotypic culture system, in vitro production of haploid male germ cells from immature human testicular tissue was also reported. Frozen-thawed 1-mm^3^ testicular fragments collected from prepubertal boys (aged 2–12 years) were cultured in a KSR-based medium supplemented with 5 IU/L of FSH, which after 16 days resulted in the formation of round spermatids. Interestingly, increasing the concentration of FSH from 5 to 50 IU/L failed to induce germ cell differentiation, perhaps due to desensitization of FSH receptors or adenyl cyclase in Sertoli cells [128]. However, it should be noted that currently such in vitro produced haploid male germ cells only serve as a proof-of-principle and would need be further analyzed for their fecundability and epigenetic characteristics before suggesting these techniques for potential clinical trials.

Using non-human primates, 3D culture models of IVS have also shown promising results. Among non-human primates, the juvenile rhesus monkey is considered a suitable model for prepubertal boys, because in both species the phase of prepubertal development is characterized by a protracted hypogonadotropic and hypoandrogenic state. These characteristics provide an ideal baseline for examining putative factors involved in the initiation of spermatogenesis. To check the efficiency of SACS and MCS culture systems in supporting the differentiation of primate germ cells, testicular cells isolated form juvenile rhesus monkeys (aged 13–33 months) were cultured in either culture system for 4–8 weeks. The MCS culture system supported the differentiation of immature testicular germ cells and after 30 days of culture, haploid male germ cells were observed [116]. However, the fertilizing potential and epigenetic characteristics of the in vitro produced haploid male germ cells were not determined.

## 8. Limitations of In Vitro Spermatogenesis in Non-Rodents

Despite the long history of experimentations targeting IVS, an optimal and efficient system capable of inducing complete differentiation of immature human testis cells or testicular tissue to form haploid male germ cells has not yet been developed. The gradual and incremental success using rodent models of IVS; however, is encouraging and suggest that recapitulation of human spermatogenesis will be eventually possible in the future. However, when such an IVS system is provided for use with human germ cells, extensive additional safety and ethical reviews would have to be completed before such tools can be considered for potential use in reproductive medicine.

Limited availability of basic knowledge about primate SSCs and their in vitro requirements, along with the long prepubertal period in primates compared with rodents, makes achieving IVS for primates even more challenging. Therefore, as a first step toward achieving complete IVS in primates, it is crucial to maintain the structural integrity of testicular tissue and extend the viability of isolated germ cells in long-term cultures. Moreover, obtaining sufficient amounts of donor primate testis tissue for various studies is difficult, if not impossible. Hence, the use of donor testes from other suitable non-rodent animal models such as neonatal pigs may provide an attractive alternative, given that pigs share considerably more anatomical and physiological similarities with humans than do rodents. Moreover, optimized in vitro and in vivo culture conditions for development of testicular cells and tissue from immature donor pigs have been developed to facilitate such studies [129,130,131,132].

Although, there are a few reports of in vitro production of haploid male germ cells in non-rodents, it has not yet been possible to produce morphologically normal, viable, motile, and fertilization-competent sperm from undifferentiated germ cells. This implies that cells at different stages of spermatogenesis have different culture requirements, which so far have not been met. Immotile in vitro produced male haploid germ cells are infertile when used in classical in vitro fertilization (IVF) or in artificial insemination (AI). Therefore, if the ultimate goal of primate IVS is to use the resultant sperm in IVF, then the current situation is still far from ideal. Additionally, even though round spermatids, elongated spermatids, or even sperm can theoretically be produced in vitro and individually used for fertilizing oocytes using intracytoplasmic injection (as discussed below), these potential haploid cells are not always suitable for forming a zygote. Therefore, even after successful development of an efficient system for primate IVS, confirmation of normalcy and fertilization-competence of the in vitro produced haploid germ cells are necessary important steps before experimental applications or potential clinical trials can be recommended.

In order to improve the in vitro culture system for non-rodents, a growing number of factors are being explored which have traditionally been neglected in the past. This includes, for example, the potential role of extracellular matrices or paracrine factors secreted by somatic components of the testis. For instance, paracrine factors secreted by Leydig and peritubular myoid cells (e.g., peritubular factor that modulates Sertoli cell function, PModS) have been shown to modulate the effects of testosterone on Sertoli cells, in turn altering the secretion of transferrin and inhibin [133]. These factors and other novel elements may play an important role in spermatogenesis; therefore, investigating the significance of putative factors is warranted and would likely help in defining optimal culture systems for achieving IVS in primates.

## 9. Future Prospects of In Vitro Spermatogenesis for Fertility Preservation and Restoration

IVS has many important potential clinical applications and in certain circumstances is the only currently conceivable safe option for preserving fertility and upholding biological fatherhood. At the present time, human IVS to allow differentiation from immature stages to fully-formed sperm has not been achieved; however, given the promising preliminary results and gradual improvements in research methodologies, this technique will hopefully be applicable in future to resolve many of the problems currently linked with the male factors of infertility.

### 9.1. Fertility Preservation of Prepubertal Cancer Patients for Subsequent Fertility Restoration

With advances in oncotherapy, thankfully >80% of childhood cancer patients survive but, due to the gonadotoxic side effects of cancer treatments, a significant number will experience subfertility or permanent sterility as adults [134,135]. Fertility has an important impact on the post-treatment quality of life. Although cryopreservation of semen prior to cancer therapy is an established method for adult male patients wishing to preserve their future fertility, semen collection is obviously not an option for pre-adolescent boys whose testes have not started sperm production. For such immature patients, cryopreservation of testicular biopsies collected prior to the start of gonadotoxic therapies is the only conceivable option to preserve their fertility potential. This is because the testis of prepubertal individuals contains SSCs, which can be potentially used to restore spermatogenesis and ultimately produce haploid germ cells [136,137].

**Figure 3:**
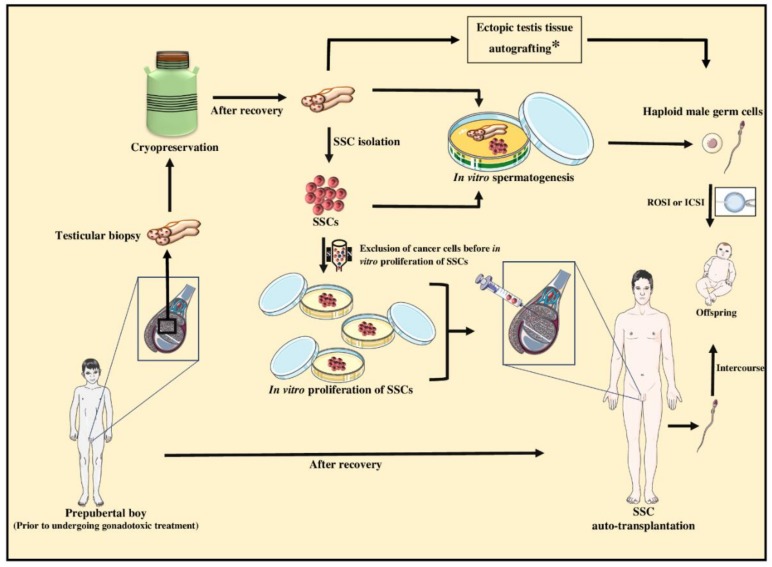
Schematic representation of potential fertility preservation options for prepubertal cancer patients. Testicular biopsies can be collected from prepubertal boys before commencing gonadotoxic cancer treatments. The testicular biopsies can be then cryopreserved as a source of spermatogonial stem cells (SSCs) in case the patient indeed become infertile. To preserve the fertility potential, different theoretical or experimental approaches using testicular biopsies or isolated SSCs can be used to produce haploid male germ cells. Advanced assisted reproductive technologies that can be considered after recovery from cancer include in vitro spermatogenesis, ectopic testicular tissue autografting, or auto-transplantation of SSCs into the donor testis to induce spermatogenesis. The resultant haploid male germ cells can be used for fertilization through natural conception or with the help of intracytoplasmic sperm injection (ICSI) or round spermatid injection (ROSI). Asterisk (*) in the boxed text is to emphasize that even though testis cells can be potentially processed to eliminate malignant cells using flow cytometry, this is not applicable with tissue fragments. Therefore, the option of autografting (testicular fragments) is only applicable if the patient was not suffering from testicular cancer or metastatic tumors due to high risk of reintroducing the pre-existing malignant cells back into the patient.

The pre-treatment testicular biopsies from immature boys can be cryopreserved and maintained in liquid nitrogen almost indefinitely, or until such time when the patients have recovered and been diagnosed with infertility as adults. Even if no sperm is found in the recovered individual’s semen, frequently haploid germ cells can still be retrieved from the testes, using established advanced reproductive technologies. This for instance includes microscopic testicular sperm extraction (micro-TESE) to be used for intracytoplasmic sperm injection (ICSI) or round-spermatid injection (ROSI). However, if retrieval of haploid germ cells is not an option, such as when there is spermatogenesis arrest at earlier stages, then the cryopreserved testicular biopsies can be thawed and used in technologies such as IVS, which hopefully by then would be optimized and proven safe. The various scenarios and theoretical options are summarized in Figure 3 and include the potential use of testis tissue fragments or isolated cell suspensions for IVS to produce haploid germ cells, followed by their use for ICSI or ROSI. Alternatively, the cryopreserved tissue fragments can be processed to obtain testis cells containing SSCs, which could include the exclusion of potentially residual malignant cells. This is then followed by subsequent expansion of SSC numbers in culture, before offering to perform auto-transplantation of SSCs for re-colonization of the individual’s testes, which can potentially lead to sufficient production of sperm to restore fertility [136,137].

Proof-of-principle studies for a number of the steps outlined in Figure 3 have been shown using various animal models, but at this time, none of the proposed approaches have been adequately tested using non-human primates to warrant even experimental use for humans. For instance, we and others have expanded the technique for male germ cell transplantation, a technique that was originally developed in mice, by showing the feasibility of autologous and homologous transplantations in various large animal models [138,139] and non-human primates, which also included successful production of fertile sperm [132]. Therefore, auto-transplantation of germ cells (containing SSCs) isolated from cryopreserved testicular biopsies is theoretically possible but not before other technical difficulties are overcome.

Firstly, it has been shown that an average testicular biopsy from prepubertal boys weighs ~31 mg and provides ~390,000 total cells, of which only ~11,700 are estimated to be spermatogonia [140]. Given the heterogenous nature of spermatogonia (as discussed above), a much smaller number of these cells would actually be expected to be functional SSCs; hence, it is unlikely that single biopsies will provide sufficient numbers of SSCs to repopulate the testis without further in vitro expansion of SSCs [140]. Therefore, an important first step toward the use of cryopreserved biopsies, as a source for auto-transplantation of testis cells, is exponential expansion of resultant SSC numbers in culture, which would take extensive additional research to optimize such an efficient culture system.

Secondly, in the process of in vitro culture, testicular cells are exposed to a number of ‘unnatural’ conditions including physical and chemical manipulations (e.g., enzymatic digestion, pH changes), and known and unknown factors (e.g., growth factors, cytokines), that may cause them to undergo oxidative stress or DNA methylation and/or histone post-translational modifications. Therefore, careful assessment of genetic and epigenetic modifications should be part of the further work on testis cell culture to ensure their safety for potential use in clinical applications [141,142,143].

Thirdly, because the biopsies are taken from immature patients before undergoing cancer treatment, they may carry malignant cells. This is especially the case for children with testicular cancer, lymphomas, leukemia, or other tumors that metastasize to the testis. In such cases, the auto-transplantation approach should not be considered due to the high risk of reintroducing malignant cells, and certainly not before the suggested methods for elimination of cancer cells using cell sorting (e.g., MACS [144]) have been optimized further and proven 100% efficient and safe [145].

Autologous grafting of the testicular biopsies has also been proposed as a potential approach for restoration of fertility in childhood cancer survivors. Auto-grafting, either in the form of orthotopic (placed within the scrotum) or ectopic (placed subcutaneously outside the scrotum), relies on the ability of the frozen-thawed immature testicular fragments in undergoing extensive development, complete differentiation, and formation of sperm or other haploid germ cells to be extracted and used in ICSI or ROSI [146]. However, because of the same reasons cited above against the use of auto-transplantation of cells, autografting of intact testicular biopsies should not be considered, if there is any chance that malignant cells may still be present in the biopsies. Nevertheless, the feasibility of auto-grafting has been shown using both ectopic and orthotopic grafting of cryopreserved immature testicular tissues in non-human primates, leading to production of sperm and even recently in the birth of a healthy monkey [146,147].

It should be noted that while the use of these testicular biopsies for xenografting into host mice has also been proposed by some fellow researchers and/or clinicians, we have deliberately refrained from including this approach as a potential option for restoration of fertility in our figures. In fact, we were first to show that fresh and cryopreserved immature testicular tissue fragments from various donor species can be grafted under the back skin of immunodeficient recipient mice where they completely develop and even produce large numbers of fertile xenogeneic sperm [148,149]. We were also among the first groups to expand the application of this testicular tissue xenografting system into primates by grafting testis tissue fragments from immature donor monkeys and mature humans [132,150,151]. Therefore, we are well-informed of the various potential applications of testicular tissue xenografting and can envision that current technical hurdles in achieving full spermatogenesis from immature human testis xenografts [152] can be sufficiently addressed. We also believe that it may even become theoretically possible to show that xenogeneic sperm from pre-treatment testicular biopsies would not carry a risk of reintroducing malignancy when used in ICSI. However, we strongly object to offering human testis tissue xenografting as a potential route to produce human sperm if it is exclusively meant to be used for human fertilization. This is because of the serious safety concerns, for instance in terms of the potential transfer of endogenous species-specific retroviral transcripts from the host mouse, as well as grave ethical and even legal limitations of using such xenogeneic sperm for human fertilization. On the other hand, if the goal is not to use the potential xenogeneic sperm for human fertilization, then further research on human testicular tissue xenografting is actually encouraged. For instance, such a system can provide a highly meritorious, novel, and unique assay for the basic research into factors that affect human testis development and spermatogenesis. It would also be a previously-unavailable tool for toxicological and pharmacological investigations into environmental toxicants or new medications that can affect human testis development or testis function [132].

Therefore, currently, the only plausibly safe approach to produce haploid male germ cells from immature human testicular biopsies for the listed potential future clinical applications is through IVS, because it does not carry a risk of reintroducing cancer cells. However, as outlined above, complete IVS has so far been only achieved using donor mice [94]. Although a similar organotypic culture system with some modifications has also been adopted to culture prepubertal human tissue, progression of spermatogonia has not been observed [153], suggesting that culture conditions developed for mouse may not directly or easily be applicable for primate IVS and would require extensive further research.

### 9.2. Upholding the Biological Fatherhood of Adult Infertile Patients

**Figure 4:**
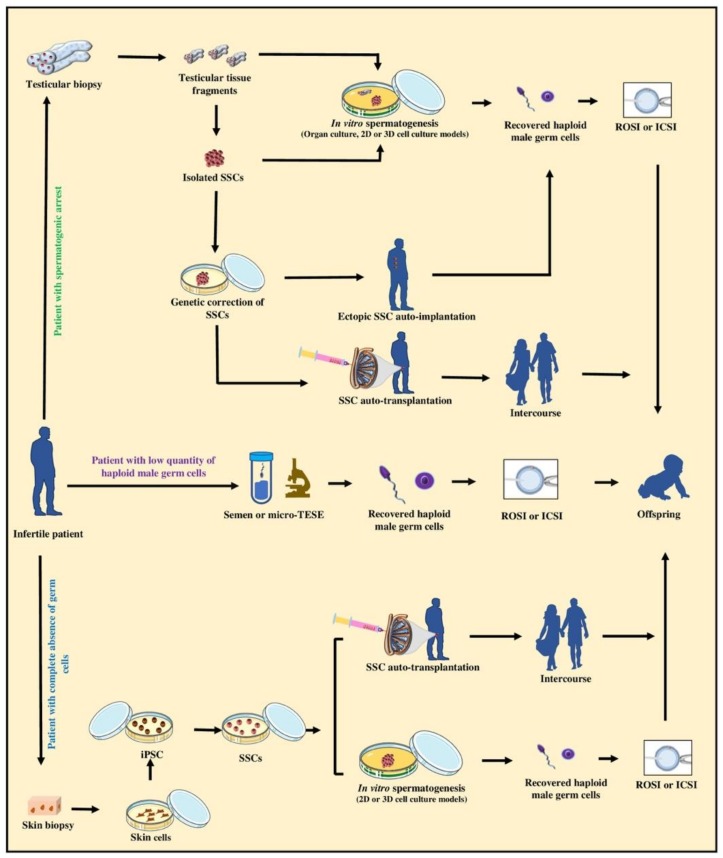
Schematic representation of standard, experimental, and theoretical options for upholding the biological fatherhood of adult infertile patients. The standard practice for upholding the biological fatherhood of an adult infertile patient is to use sperm from ejaculated semen or to use testicular sperm obtained by microsurgical extraction (TESE) for in vitro fertilization such as using intracytoplasmic sperm injection (ICSI) or round spermatid injection (ROSI). However, this option is applicable only when the patient’s testis contains haploid male germ cells, such as in men with obstructive azoospermia. When infertility is due to spermatogenic arrest, testicular biopsies containing SSCs can be potentially obtained and used either for in vitro spermatogenesis (i.e., organotypic culture, 2D, or 3D cell culture models) in optimized culture conditions to produce haploid male germ cells. Moreover, theoretically genetic correction of isolated SSCs can also be applied in vitro before SSCs can be used for ectopic SSC auto-implantation or auto-transplantation into the patient testis to regenerate spermatogenesis and possibly lead to natural fertility. If the testis of the infertile patients does not contain normal germ cells, skin biopsies can be collected from the patient to theoretically produce induced pluripotent stem cells (iPSC), followed by differentiation of iPSC into SSCs. Such SSCs can then be potentially used for in vitro spermatogenesis (i.e., 2D or 3D cell culture models) to produce haploid male germ cells that can be used to fertilize eggs by ICSI or ROSI. Alternatively, auto-transplantation of SSCs can be potentially used to regenerate spermatogenesis and possibly natural fertility. It is important to emphasize that extensive further research is required to fully examine the various technical, safety, ethical, and legal aspects of the proposed experimental and theoretical options depicted here before they can be recommended for experimental or potential clinical trials.

Meta-analysis of infertility data from various countries and continents concludes that globally an estimated 15% of couples experience infertility. Although the rate differs in different regions, overall about half (range 20%–70%) of the cases are thought to be due to the male factor [154]. Infertility in male also seems to show an uptrend, which can be due to various reasons including varicoceles, medications, obstruction, genetic disorders, or exposure to known and unknown environmental toxicants [155]. A number of infertility cases can be treated using experimental or clinical assisted reproductive techniques, as schematically shown in Figure 4. The currently applicable options include the use of ICSI for men with low numbers of sperm or the use of ICSI or ROSI following microsurgery for testicular sperm or spermatid extractions (micro-TESE) from men with obstructive azoospermia. However, in cases where the patient’s testis does not contain haploid male germ cells, these latter techniques cannot be applied.

Patients lacking haploid germ cells in the testis can be divided into those with complete absence of germ cells and those with meiotic-stage, or pre-meiotic spermatogenic arrest [156]. At times, the spermatogenic arrest is treatable using conventional andrology treatments and interventions such as hormonal therapy. To uphold the biological fatherhood of the remaining patients, however, the options are currently limited and novel biotechnology approaches may need to be developed or considered in the future. These theoretical proposed options include the use of testis tissue biopsies or cells in organotypic tissue culture, or 2D/3D cell suspension culture systems, as outlined above. Using such culture systems, the cause of the spermatogenetic arrest can also be explored further and potentially may even be overcome for instance by supplying the missing factors [95] to produce haploid germ cells in vitro for subsequent use in ICSI or ROSI.

Alternatively, testicular biopsies from patients who inherently have very few germ cells can be used as a source of SSCs to be expanded in vitro, followed by auto-transplantation to colonize their testes, and potentially initiate spermatogenesis to allow natural fertility. Theoretically, resultant SSCs can also be implanted ectopically under the skin of the patient for regeneration of testicular tissue to provide haploid germ cells for microsurgical extraction from the regenerated implants, followed by ICSI or ROSI. We and other have shown that testis cells collected after enzymatic digestion of immature donors of various species indeed maintain an unexpected de novo organogenic potential to regenerate functional testis tissue when cell aggregates are implanted under the skin of recipient animal models [132,157]. Interestingly, to overcome spermatogenic arrest at the level of meiotic cells using a mouse model, secondary spermatocytes have been injected into oocytes for the production of viable embryos and even offspring [158]. Intracytoplasmic injection of secondary spermatocytes has also been applied in human; however, the efficiency was reported to be low [159].

Yet, in situations where the patient’s testis does not support the differentiation of germ cells or does not have any type of germ cells due to genetic or chromosomal mutations, at least theoretically, they can still be a candidate for future potential interventions. This includes the in vitro use of gene therapy or genetic correction techniques to overcome the spermatogenic arrest if in the future safe genome editing tools become available. Proof-of-principle studies using a mouse model of infertility have shown the feasibility of such approaches. For instance, due to a mutation of the c-kit ligand (KITL), spermatogenesis is completely arrested and only few abnormal SSCs are present in the testes of Steel (*Sl*) mice. However, providing the missing component, in the form of recombinant KIT, to the cultured testicular fragments resulted in a dose-dependent increase in the number of SSCs and ultimately in complete IVS [95]. Emergence of induced pluripotent stem cells (iPSC) from somatic cells, which can also be driven to form germ cells, has opened new possibilities. Although the iPSC technology in its current form cannot be used in humans, if similar but safe future alternative technologies become available, theoretically they may provide another unique opportunity to allow fatherhood for individuals who have no other options. Such newly-derived SSCs may be used in IVS or in autologous transplantation to allow parenthood (Figure 4).

## 10. Conclusions

SSCs are uniquely positioned among adult stem cells because of their potential for passing on genes to the progeny. However, our current knowledge of their biology is limited and so is our ability to harness their full potential for in vitro and in vivo applications in assisted reproductive technologies. Recent progress in organotypic testicular tissue culture as well 2D and 3D cell suspension culture systems may eventually lead to complete IVS using human testicular biopsies to allow preservation and restoration of fertility potential of prepubertal cancer patients undergoing gonadotoxic treatment. Moreover, such optimal IVS systems may also offer unprecedented new perspectives in the study of infertility causes and even approaches to overcome some of the currently untreatable infertility cases in men. However, it has to be emphasized that the proposed potential options discussed here are purely theoretical or are still at early experimental stages, and any recommendation for applying them in the future would require extensive safety, feasibility, and bioethical considerations.

## Figures and Tables

**Table 1:** Molecular markers of undifferentiated spermatogonia in primates and mice.

Markers	Mouse	Monkey	Human
**Cell Surface Markers**
THY1 (CD90)	+ [30]	+ [44]	+ [32]
β1-integrin (ITGB1, CD29)	+ [29]	+ [45]	+ [46]
α6-Integrin (ITGA6, CD49f)	+ [29]	+ [44]	+ [32]
PROM1 (CD133)	?	?	+ [32]
GFRα1	+ [47]	+ [28]	+ [32]
CDH1	+ [39]	?	?
SSEA4	?	+ [44]	+ [48]
FGFR3	+ [49]	?	+ [50]
DSG2	?	?	+ [50]
LPPR3	?	?	+ [51]
TSPAN33	?	?	+ [51]
CD9	+ [31]	?	+ [52]
GPR125	+ [35]	?	+ [36]
**Intracellular Markers**
PAX7	+ [53]	+ [53]	+ [53]
DDX4 (VASA)	+ [54]	+ [25]	+ [55]
DAZL	+ [13]	+ [25]	+ [33]
CHK2	?	?	+ [43]
LIN28	+ [22]	+ [23]	+ [23]
MAGEA4	?	+ [56]	+ [57]
NANOS2	+ [58]	+ [59]	+ [60]
NANOS3	+ [60]	+ [59]	+ [60]
SALL4	+ [61]	+ [27]	+ [24]
UCHL1 (PGP9.5)	+ [62]	+ [24]	+ [33]
PLZF	+ [63]	+ [25]	+ [33]
POU5F1 (OCT4)	+ [64]	+ [56]	+ [65]
ID4	+ [34]	?	+ [36]
NEUROG3 (NGN3)	+ [66]	+ [28]	?
DMRT1	+ [67]	?	+ [68]
UTF1	+ [69]	+ [23]	+ [70,71]
TCF3	?	?	+ [33]
SPOCD1	?	?	+ [68]
ENO2	?	+ [72]	+ [70]
SNAP91	?	?	+ [50]
CBL	?	?	+ [50]
PIWIL4	?	?	+ [51]
ZKSCAN2	?	?	+ [68]
RET	+ [37]	?	?
STRA8	+ [38]	?	?
RBM	+ [73]	?	+ [74]
TSPY	− [42]	?	+ [41]

“+” or “−“ indicate that evidence is available on the presence or absence of these markers, respectively; “?” indicates that we are not aware of studies that have examined these markers.

**Table 2:** Culture conditions for long-term in vitro maintenance of rodent or primate spermatogonial stem cells (SSCs).

Culture Conditions	Mouse			Monkey *	Human		
Donor age	Newborn	4.5–7.5-day-old	8-day-old	3.7–4.6 years (average age)	NA	14, 34, and 45 years	22–35 years
SSC isolation method	Two-step enzymatic digestion	Two-step enzymatic digestion	Two-step enzymatic digestion	Two-step enzymatic digestion	Two-step enzymatic digestion	Two-step enzymatic digestion	Two-step enzymatic digestion
SSC enrichment method	Diff. plat.	MACS	MACS	Diff. plat./un enriched	Diff. plat.	Diff. plat. + MACS	Diff. plat. + MACS
Basic medium	StemPro-34 SFM	Alpha-MEM	StemPro-34 SFM	Alpha-MEM	StemPro-34 SFM	StemPro-34 SFM	StemPro-34 SFM
Additional supplements	StemPro supplement	Supplement	StemPro supplement	NA	StemPro supplement	StemPro supplement	StemPro supplement
Protein/serum source	FCS	BSA	Albumax II	FBS	FCS	BSA	FBS, BSA
Growth factors	GDNF, bFGF, EGF, LIF	GDNF, bFGF, GFRα1	GDNF, bFGF, EGF	NA	GDNF, LIF, EGF, bFGF	GDNF, LIF, EGF, bFGF	GDNF, LIF, EGF, bFGF
Feeder cells	MEF	STO	NA	NA/Somatic cells	NA	NA	NA
Temperature	37 ^o^C	37 ^o^C	37 ^o^C	35 ^o^C	37 ^o^C	37 ^o^C	37 ^o^C
Subculture	10–14 days	5–7 days	5–6 days	NA	7–10 days	10–15 days	NA
Max. culture duration	∼160 days	~180 days	~178 days	~11 days	~196 days	~120 days	~60 days
Germ cell clump formation	Yes	Yes	Yes	Yes	Yes	Yes	Yes
SSC identification	Flow cytometry	Immunostaining	Flow cytometry	Immunostaining	Immunostaining	PCR	Immunostaining
Functional analyses	Yes	Yes	Yes	Yes	Yes	No	No
Reference	[108]	[109]	[110]	[111]	[83]	[112]	[82]

* We are unaware of reports of longer SSC cultivation times than 11 days for non-human primates.

## Abbreviations

**Definition**	**Abbreviation**
Artificial insemination	AI
Cadherin-1	CDH1
Casitas B-lineage lymphoma	CBL
Checkpoint kinase 2	CHK2
c-Kit ligand	KITL
Cluster of differentiation 9	CD9
Cluster of differentiation 90	CD90
Days post-coitum	dpc
Days post-partum	dpp
DEAD-box helicase 4	DDX4
Deleted in azoospermia-like	DAZL
Desmoglein 2	DSG2
Developmental pluripotency associated 4	DPPA4
Doublesex and mab-3 related transcription factor 1	DMRT1
Eagle’s minimum essential media	MEM
Embryonic stem	ES
Engineered blood-testis barrier	eBTB
Enolase 2	ENO2
Extracellular matrices	ECM
Fetal bovine serum	FBS
Fibroblast growth factor receptor 3	FGFR3
Fluorescence in situ hybridization	FISH
Fluorescence-activated cell sorting	FACS
GDNF family receptor alpha-1	GFRα1
G-protein coupled receptor 125	GPR125
In vitro fertilization	IVF
In vitro spermatogenesis	IVS
Induced pluripotent stem cells	iPSC
Intracytoplasmic sperm injection	ICSI
Knockout serum replacement	KSR
Lin-28 homology A	LIN28
Magnetic-activated cell sorting	MACS
Melanoma-associated antigen 4	MAGEA4
Methylcellulose	MCS
Microscopic testicular sperm extraction	micro-TESE
Nanos C2HC-type zinc finger	NANOS
Neurogenin 3	NGN3
Octamer-binding transcription factor 4	OCT4
Paired box 7	PAX7
Peritubular factor that modulates Sertoli cell function	PModS
Phospholipid phosphatase related 3	PLPPR3
Piwi like RNA-mediated gene silencing 4	PIWIL4
POU class 5 homeobox 1	POU5F1
Primordial germ cells	PGCs
Prominin 1 (Cluster of differentiation 133)	PROM1 (CD133)
Promyelocytic leukemia zinc finger protein	PLZF
Ret proto-oncogene	RET
Retinoic acid	RA
RNA binding motif	RBM
Round-spermatid injection	ROSI
Sal-like protein 4	SALL4
Serum free medium	SFM
Soft-agar culture system	SACS
Spermatogonial stem cells	SSCs
SPOC domain containing 1	SPOCD1
Stage-specific embryonic antigen-4	SSEA4
Steel	SL
Stimulated by retinoic acid 8	STRA8
Synaptosome associated protein 91	SNAP91
Testis-specific Y-encoded protein	TSPY
Tetraspanin 33	TSPAN33
Three-dimensional	3D
Three-layer gradient system	3-LGS
Transformer-1	TRA-1
Two-dimensional	2D
Ubiquitin C-terminal hydrolase L1	UCHL1
Undifferentiated embryonic cell transcription factor 1	UTF1
Vasa gene-encoded RNA binding protein	VASA
Zinc finger with KRAB and SCAN domains 2	ZKSCAN2
